# A New Stochastic Model Updating Method Based on Improved Cross-Model Cross-Mode Technique

**DOI:** 10.3390/s21093290

**Published:** 2021-05-10

**Authors:** Hui Chen, Bin Huang, Kong Fah Tee, Bo Lu

**Affiliations:** 1 School of Civil Engineering & Architecture, Wuhan University of Technology, Wuhan 430070, China; huichenvip@163.com (H.C.); lubo@whut.edu.cn (B.L.); 2 Wuhan Institute of Technology, College of Post and Telecommunication, Wuhan 430074, China; 3 School of Engineering, University of Greenwich, Kent ME4 4TB, UK; K.F.Tee@greenwich.ac.uk

**Keywords:** stochastic model updating, stochastic hybrid perturbation-Galerkin method, cross-model cross-mode method

## Abstract

This paper proposes a new stochastic model updating method to update structural models based on the improved cross-model cross-mode (ICMCM) technique. This new method combines the stochastic hybrid perturbation-Galerkin method with the ICMCM method to solve the model updating problems with limited measurement data and uncertain measurement errors. First, using the ICMCM technique, a new stochastic model updating equation with an updated coefficient vector is established by considering the uncertain measured modal data. Then, the stochastic model updating equation is solved by the stochastic hybrid perturbation-Galerkin method so as to obtain the random updated coefficient vector. Following that, the statistical characteristics of the updated coefficients can be determined. Numerical results of a continuous beam show that the proposed method can effectively cope with relatively large uncertainty in measured data, and the computational efficiency of this new method is several orders of magnitude higher than that of the Monte Carlo simulation method. When considering the rank deficiency, the proposed stochastic ICMCM method can achieve more accurate updating results compared with the cross-model cross-mode (CMCM) method. An experimental example shows that the new method can effectively update the structural stiffness and mass, and the statistics of the frequencies of the updated model are consistent with the measured results, which ensures that the updated coefficients are of practical significance.

## 1. Introduction

As an important numerical method, the finite element (FE) modeling is widely used in engineering. However, due to the uncertain influence of many factors such as material properties, modeling, construction and so on, there are often some differences between the FE model and the real structure. Therefore, model updating is used to update the structural FE model to make a better match between the dynamic responses of the analytical model and that of the actual structure. In this way, an accurate and reliable model can be obtained, and further can be applied to structural health monitoring, optimization design or reliability analysis.

At present, the updating of FE model is mainly to adjust the stiffness matrix and mass matrix in the FE model, or to update the physical parameters in these matrices, so that the updated model can be used to reasonably predict the structural real mechanical behaviors. By now, many deterministic model updating methods have been developed, which mainly include optimization based model updating methods [1,2,3,4,5,6,7], or FE based model updating methods [8,9,10,11,12], Bayesian model updating methods and recently developed model updating methods based on artificial intelligence [13,14,15].

It is found that the model updating methods based on dynamic measurement data attracted more and more attention in recent decades [16]. Many researchers have carried out extensive research in this area and achieved a lot of research results [17,18,19]. Among these methods, a CMCM model updating method was developed by Hu and Li [9]. Different from the traditional model updating methods, this method can update the stiffness, mass and damping matrix at the same time. Moreover, this method is a non-iterative method, so it is very cost-effective in computational time. In the CMCM method, by multiplying the measured modes with the calculated modes of the structure, a number of updated equations can be constructed by using only few measured modes. Based on the modal expansion and model simplification technique, Li [20] proposed a new CMCM method for damage detection using incomplete modal data. Wang et al. [21] used the CMCM method to conduct an experimental study of an offshore platform. These studies confirm the effectiveness of the CMCM method when the actual structural measurement modes are incomplete and only low-order measurement modes are available. Based on the existing CMCM method, Liu [22] proposed the ICMCM method, which makes full use of measurement data to further increase the number of updating equations. However, these CMCM methods are only involved in deterministic FE model updating. When the uncertainty of structural models is unavoidable or the measurement noise is considered, the existing CMCM methods are not available for model updating. Therefore, it is very significant to improve the deterministic CMCM methods, especially the proposed ICMCM method, to update structural models.

In regard to stochastic model updating, the Monte-Carlo simulation methods [23], perturbation methods [2,24] and Bayesian methods [25,26,27,28] are widely used. In [23], the Monte-Carlo simulation with a large number of samples is used to calculate the statistical characteristics of updated parameters, but for large structures such as offshore platforms, the calculation is too time-consuming to accept. Unlike the Monte-Carlo simulation method, the perturbation method has the advantages of simple derivation and high computational efficiency, but it is limited by small variability. Based on the Markov chain Monte Carlo sampling, the Bayesian methods are another significant selection for stochastic model updating, but it seems to be a deficiency for the Bayesian methods, which is that the time-consuming repeated FE calculation is needed. To improve the calculation efficiency, Wan [28] and Fang [29] improved the original Bayesian methods by a Gaussian surrogate model and a stochastic response surface model, respectively. Different from the above-mentioned stochastic model updating methods, Huang et al. [12] recently proposed a hybrid perturbation-Galerkin model updating method (HPG), which is of high accuracy and efficiency for model updating. This method provided a new idea and complete frame for extending the deterministic model updating methods to the stochastic domain.

Inspired by the idea of HPG, this paper combines the stochastic hybrid perturbation-Galerkin method with the ICMCM method to update structural models. In this way, the deterministic ICMCM method is extended to a stochastic field. Then the effectiveness of this proposed method is verified by a numerical example of a two-span continuous beam, where limited measurement data and uncertain measurement errors are considered. The updating results of an experimental beam show that the new method can effectively update the stiffness and mass of the beam, and the statistical results of the updated frequencies are consistent with the measured results.

## 2. Theoretical Background

### 2.1. The ICMCM Method

Here the deterministic ICMCM method is shortly reviewed. Considering an undamped structure with *N* degrees of freedom, the eigenvalues and eigenvectors of the initial structural model satisfy the following equation, as

(1) $$K\Phi_{i}=\lambda_{i}M\Phi_{i}\;(i=1,\;\ldots ,\;N_{c})$$

where **K** and **M** are the global stiffness matrix and mass matrix of the initial structural model, respectively, and $\lambda_{i}$ and $\Phi_{i}$ represent the *i*th-order eigenvalue and eigenvector of the initial model, respectively. *N_c_* is the number of calculated eigenvalues and eigenvectors of the initial model.

With respect to the real structure, its eigenvalue equation can be written as

(2) $$K^{*}\Phi_{j}^{*}=\lambda_{j}^{*}M^{*}\Phi_{j}^{*}\;(j=1,\;\ldots ,\;N_{m})$$

where $K^{*}$ and $M^{*}$ are the global stiffness matrix and mass matrix of the real structure, respectively. $\lambda_{j}^{*}$ and $\Phi_{j}^{*}$ represent the *j*th order eigenvalue and eigenvector of the real structure, respectively. *N_m_* is the number of eigenvalues and eigenvectors of the real structure.

The relationship between the stiffness and mass matrices of the real structure and those of the initial model can be written, respectively, as

(3) $$K^{*}=K+\sum_{n=1}^{N_{e}}\alpha_{n}K_{n}$$

(4) $$M^{*}=M+\sum_{n=1}^{N_{e}}\beta_{n}M_{n}$$

where $N_{e}$ is the total number of structural elements. $K_{n}$ and $M_{n}$ are *N* × *N* dimensional expanding matrices of stiffness and mass matrices of the *n*th element, respectively, where other parts are zeroes. $\alpha_{n}$ and $\beta_{n}$ are the variations of the real stiffness matrix and mass matrix of the *n*th element of the structure relative to those of the initial model, respectively, which are named as updated coefficients.

Premutiplying Equation (1) by ${\left(\Phi_{j}^{*}\right)}^{T}$ (*j* = 1, ..., *N_m_*) and Equation (2) by ${\left(\Phi_{i}\right)}^{T}$ (*i* = 1, ..., *N_c_*), respectively, there are

(5) $${\left(\Phi_{j}^{*}\right)}^{T}K\Phi_{i}=\lambda_{i}{\left(\Phi_{j}^{*}\right)}^{T}M\Phi_{i}$$

(6) $${\left(\Phi_{i}\right)}^{T}K^{*}\Phi_{j}^{*}=\lambda_{j}^{*}{\left(\Phi_{i}\right)}^{T}M^{*}\Phi_{j}^{*}$$

Dividing Equation (6) by the transposed Equation (5) yields

(7) $$\frac{{\left(\Phi_{i}\right)}^{T}K^{*}\Phi_{j}^{*}}{{\left(\Phi_{i}\right)}^{T}K\Phi_{j}^{*}}=\frac{\lambda_{j}^{*}}{\lambda_{i}}\frac{{\left(\Phi_{i}\right)}^{T}M^{*}\Phi_{j}^{*}}{{\left(\Phi_{i}\right)}^{T}M\Phi_{j}^{*}}$$

Then, substituting Equations (3) and (4) into Equation (7), one has

(8) $$1+\sum_{n=1}^{N_{e}}\alpha_{n}\frac{{\left(\Phi_{i}\right)}^{T}K_{n}\Phi_{j}^{*}}{{\left(\Phi_{i}\right)}^{T}K\Phi_{j}^{*}}=\frac{\lambda_{j}^{*}}{\lambda_{i}}\left(1+\sum_{n=1}^{N_{e}}\beta_{n}\frac{{\left(\Phi_{i}\right)}^{T}M_{n}\Phi_{j}^{*}}{{\left(\Phi_{i}\right)}^{T}M\Phi_{j}^{*}}\right)$$

Further Equation (8) can be changed to

(9) $$\sum_{n=1}^{N_{e}}\alpha_{n}\frac{{\left(\Phi_{i}\right)}^{T}K_{n}\Phi_{j}^{*}}{{\left(\Phi_{i}\right)}^{T}K\Phi_{j}^{*}}-\sum_{n=1}^{N_{e}}\beta_{n}\frac{\lambda_{j}^{*}}{\lambda_{i}}\frac{{\left(\Phi_{i}\right)}^{T}M_{n}\Phi_{j}^{*}}{{\left(\Phi_{i}\right)}^{T}M\Phi_{j}^{*}}=\frac{\lambda_{j}^{*}}{\lambda_{i}}-1$$

Through solving Equation (9), one can obtain the updated coefficients $\alpha_{n}$ and $\beta_{n}$. The above process is referred to as the CMCM method in [9]. However, since only the first few modes can be accurately measured in actual modal tests, the dimension of Equation (9) may be much less than the number of updated coefficients, which often leads to inaccurate updating results. Therefore, as an improvement of the CMCM method, the ICMCM method is proposed in [22] to increase the number of updating equations, which is illustrated in the following.

Premutiplying Equation (2) by $\Phi_{i}^{*}$ yields

(10) $${\left(\Phi_{i}^{*}\right)}^{T}K^{*}\Phi_{j}^{*}=\lambda_{j}^{*}{\left(\Phi_{i}^{*}\right)}^{T}M^{*}\Phi_{j}^{*}$$

Substituting Equations (3) and (4) into Equation (10), one can have

(11) $${\left(\Phi_{i}^{*}\right)}^{T}\left(K+\sum_{n=1}^{N_{e}}\alpha_{n}K_{n}\right)\Phi_{j}^{*}=\lambda_{j}^{*}{\left(\Phi_{i}^{*}\right)}^{T}\left(M+\sum_{n=1}^{N_{e}}\beta_{n}M_{n}\right)\Phi_{j}^{*}$$

Afterwards according to Equation (11), there is

(12) $$\sum_{n=1}^{N_{e}}\alpha_{n}{\left(\Phi_{i}^{*}\right)}^{T}K_{n}\Phi_{j}^{*}+\sum_{n=1}^{N_{e}}\beta_{n}{\left(\Phi_{i}^{*}\right)}^{T}M_{n}\Phi_{j}^{*}=\lambda_{j}^{*}{\left(\Phi_{i}^{*}\right)}^{T}M\Phi_{j}^{*}-{\left(\Phi_{i}^{*}\right)}^{T}K\Phi_{j}^{*}$$

It is observed that Equations (9) and (12) have the same updated coefficients. In this way, more dimensional updating equations are constructed.

### 2.2. The Stochastic ICMCM Method

In the process of modal tests of real structures, the measurement errors are inevitable and they can be assumed as random. It is assumed that the *j*th measurement eigenpair ($\lambda_{j}^{*}$, $\Phi_{j}^{*}$) (*j* = 1, 2, …) are expressed, respectively, as

(13) $$\lambda_{j}^{*}=\lambda_{0j}^{*}+\xi_{j}\lambda_{1j}^{*}$$

(14) $$\Phi_{j}^{*}=\Phi_{0j}^{*}+\xi_{j}\Phi_{1j}^{*}$$

where $\lambda_{0j}^{*}$ and $\Phi_{0j}^{*}$ are the mean values of the *j*th measured eigenpair, respectively. $\lambda_{1j}^{*}$ and $\Phi_{1j}^{*}$ are the deterministic parts of measurement errors of the *j*th eigenpair, respectively. $\xi_{j}$ are the random variables related to the measurement errors. The distribution types of the random variables are determined by true measurement data or experience.

Given that all random variables $\xi_{j}$ are completely correlated and are represented as a random variable ξ, the updated coefficients of the stiffness and mass matrices of the *n*th element are expanded by using power series expansion, as

(15) $$\alpha_{n}\left(\xi \right)=\alpha_{n0}^{}+\alpha_{n1}^{}\xi +\alpha_{n2}^{}\xi^{2}+\alpha_{n3}^{}\xi^{3}+\cdots$$

(16) $$\beta_{n}\left(\xi \right)=\beta_{n0}^{}+\beta_{n1}^{}\xi +\beta_{n2}^{}\xi^{2}+\beta_{n3}^{}\xi^{3}+\cdots$$

where $\alpha_{n1}^{}$ (*i* = 1, 2, …) and $\beta_{n1}^{}$ (*i* = 1, 2, …) are the deterministic coefficients of the expansions in Equation (15) and Equation (16), respectively.

Taking into account that $\xi_{j}=\xi$, and substituting Equations (13)–(16) into Equations (9) and (12), respectively, one can have

(17) $$\begin{array}{l} \sum_{n=1}^{N_{e}}\left(\alpha_{n0}^{}+\alpha_{n1}^{}\xi +\alpha_{n2}^{}\xi^{2}+\alpha_{n3}^{}\xi^{3}+\cdots \right){\left(\Phi_{i}\right)}^{T}K_{n}\left(\Phi_{0j}^{*}+\xi \Phi_{1j}^{*}\right)\\ +\sum_{n=1}^{N_{e}}-\left(\beta_{n0}^{}+\beta_{n1}^{}\xi +\beta_{n2}^{}\xi^{2}+\beta_{n3}^{}\xi^{3}+\cdots \right)\lambda_{0j}^{*}{\left(\Phi_{i}\right)}^{T}M_{n}\left(\Phi_{0j}^{*}+\xi \Phi_{1j}^{*}\right)\\ =\left(\lambda_{0j}^{*}+\xi \lambda_{1j}^{*}\right){\left(\Phi_{i}\right)}^{T}M\left(\Phi_{0j}^{*}+\xi \Phi_{1j}^{*}\right)-{\left(\Phi_{i}\right)}^{T}K\left(\Phi_{0j}^{*}+\xi \Phi_{1j}^{*}\right) \end{array}$$

(18) $$\begin{array}{l} \sum_{n=1}^{N_{e}}\left(\alpha_{n0}^{}+\alpha_{n1}^{}\xi +\alpha_{n2}^{}\xi^{2}+\alpha_{n3}^{}\xi^{3}+\cdots \right){\left(\Phi_{0i}^{*}+\xi \Phi_{1i}^{*}\right)}^{T}K_{n}\left(\Phi_{0j}^{*}+\xi \Phi_{1j}^{*}\right)\\ +\sum_{n=1}^{N_{e}}\left(\beta_{n0}^{}+\beta_{n1}^{}\xi +\beta_{n2}^{}\xi^{2}+\beta_{n3}^{}\xi^{3}+\cdots \right)\left(\lambda_{0j}^{*}+\xi \lambda_{1j}^{*}\right){\left(\Phi_{0i}^{*}+\xi \Phi_{1i}^{*}\right)}^{T}M_{n}\left(\Phi_{0j}^{*}+\xi \Phi_{1j}^{*}\right)\\ =\left(\lambda_{0j}^{*}+\xi \lambda_{1j}^{*}\right){\left(\Phi_{0i}^{*}+\xi \Phi_{1i}^{*}\right)}^{T}M\left(\Phi_{0j}^{*}+\xi \Phi_{1j}^{*}\right)-{\left(\Phi_{0i}^{*}+\xi \Phi_{1i}^{*}\right)}^{T}K\left(\Phi_{0j}^{*}+\xi \Phi_{1j}^{*}\right) \end{array}$$

It is obvious that Equations (17) and (18) are stochastic algebraic equations with unknown coefficients $\alpha_{n1}^{}$ (*i* = 1, 2, …) and $\beta_{n1}^{}$ (*i* = 1, 2, …). To solve the unknown coefficients in Equations (17) and (18), the HPG method is adopted. First, the higher-order perturbation method is used to obtain the expansion coefficients $\alpha_{n1}^{}$ and $\beta_{n1}^{}$ (*i* = 1, 2, …), which correspond to the power polynomial basis 1, ξ, $\xi^{2}$, $\xi^{3}$… in Equations (1) and (16).

Considering the non-zero order polynomials related to Equation (17) and Equation (18), respectively, there are

(19) $$\sum_{n=1}^{N_{e}}\alpha_{n0}^{}{\left(\Phi_{i}\right)}^{T}K_{n}\Phi_{0j}^{*}+\sum_{n=1}^{N_{e}}-\lambda_{0j}^{*}\beta_{n0}^{}{\left(\Phi_{i}\right)}^{T}M_{n}\Phi_{0j}^{*}=\lambda_{0j}^{*}{\left(\Phi_{i}\right)}^{T}M\Phi_{0j}^{*}-{\left(\Phi_{i}\right)}^{T}K\Phi_{0j}^{*}$$

(20) $$\sum_{n=1}^{N_{e}}\alpha_{n0}^{}{\left(\Phi_{0i}^{*}\right)}^{T}K_{n}\Phi_{0j}^{*}+\sum_{n=1}^{N_{e}}\beta_{n0}^{}{\left(\Phi_{0i}^{*}\right)}^{T}M_{n}\Phi_{0j}^{*}=\lambda_{0j}^{*}{\left(\Phi_{0i}^{*}\right)}^{T}M\Phi_{0j}^{*}-{\left(\Phi_{0i}^{*}\right)}^{T}K\Phi_{0j}^{*}$$

Further Equations (19) and (20) are rewritten as compact matrices, as

(21) $$\left[\begin{array}{cc} C_{}^{{}^{\left(0\right)}} & E_{}^{{}^{\left(0\right)}} \end{array}\right]\cdot \gamma^{\left(0\right)}=f_{}^{{}^{\left(0\right)}}$$

(22) $$\left[\begin{array}{cc} C_{†}^{{}^{\left(0\right)}} & E_{†}^{{}^{\left(0\right)}} \end{array}\right]\cdot \gamma^{\left(0\right)}=f_{†}^{{}^{\left(0\right)}}$$

where $C^{\left(0\right)}={\left(\Phi_{i}\right)}^{T}K_{n}\Phi_{0j}^{*}$, $E^{\left(0\right)}=\sum_{n=1}^{N_{e}}-\lambda_{0j}^{*}{\left(\Phi_{i}\right)}^{T}M_{n}\Phi_{0j}^{*}$, $C_{†}^{{}^{\left(0\right)}}={\left(\Phi_{0i}^{*}\right)}^{T}K_{n}\Phi_{0j}^{*}$, $E_{†}^{{}^{\left(0\right)}}=-\lambda_{0j}^{*}{\left(\Phi_{0i}^{*}\right)}^{T}M_{n}\Phi_{0j}^{*}$, $f^{\left(0\right)}=\lambda_{0j}^{*}{\left(\Phi_{i}\right)}^{T}M\Phi_{0j}^{*}-{\left(\Phi_{i}\right)}^{T}K\Phi_{0j}^{*}$, $f_{†}^{{}^{\left(0\right)}}=\lambda_{0j}^{*}{\left(\Phi_{0i}^{*}\right)}^{T}M\Phi_{0j}^{*}-{\left(\Phi_{0i}^{*}\right)}^{T}K\Phi_{0j}^{*}$ and $\gamma^{\left(0\right)}={\left[\begin{array}{cc} \alpha^{\left(0\right)} & \beta^{\left(0\right)} \end{array}\right]}^{T}$.

Combining Equation (21) with Equation (22), there is

(23) $$\left[\begin{array}{cc} C_{I}^{{}^{\left(0\right)}} & E_{I}^{{}^{\left(0\right)}} \end{array}\right]\cdot \gamma^{\left(0\right)}=f_{I}^{{}^{\left(0\right)}}$$

where $C_{I}^{{}^{\left(0\right)}}=\left[\begin{array}{c} C^{\left(0\right)}\\ C_{†}^{{}^{\left(0\right)}} \end{array}\right]$, $E_{I}^{{}^{\left(0\right)}}=\left[\begin{array}{c} E^{\left(0\right)}\\ E_{†}^{{}^{\left(0\right)}} \end{array}\right]$ and $f_{I}^{{}^{\left(0\right)}}=\left[\begin{array}{c} f_{}^{{}^{\left(0\right)}}\\ f_{†}^{{}^{\left(0\right)}} \end{array}\right]$.

For the first-order power polynomial base ξ, correspondingly, there are

(24) $$\begin{array}{l} \sum_{n=1}^{N_{e}}\left(\alpha_{n0}^{}+\alpha_{n1}^{}\xi \right){\left(\Phi_{i}\right)}^{T}K_{n}\left(\Phi_{0j}^{*}+\xi \Phi_{1j}^{*}\right)+\sum_{n=1}^{N_{e}}-\left(\beta_{n0}^{}+\beta_{n1}^{}\xi \right)\lambda_{0j}^{*}{\left(\Phi_{i}\right)}^{T}M_{n}\left(\Phi_{0j}^{*}+\xi \Phi_{1j}^{*}\right)\\ =\left(\lambda_{0j}^{*}+\xi \lambda_{1j}^{*}\right){\left(\Phi_{i}\right)}^{T}M\left(\Phi_{0j}^{*}+\xi \Phi_{1j}^{*}\right)-{\left(\Phi_{i}\right)}^{T}K\left(\Phi_{0j}^{*}+\xi \Phi_{1j}^{*}\right) \end{array}$$

(25) $$\begin{array}{l} \sum_{n=1}^{N_{e}}\left(\alpha_{n0}^{}+\alpha_{n1}^{}\xi \right){\left(\Phi_{0i}^{*}+\xi \Phi_{1i}^{*}\right)}^{T}K_{n}\left(\Phi_{0j}^{*}+\xi \Phi_{1j}^{*}\right)+\sum_{n=1}^{N_{e}}\left(\beta_{n0}^{}+\beta_{n1}^{}\xi \right)\left(\lambda_{0j}^{*}+\xi \lambda_{1j}^{*}\right){\left(\Phi_{0i}^{*}+\xi \Phi_{1i}^{*}\right)}^{T}M_{n}\left(\Phi_{0j}^{*}+\xi \Phi_{1j}^{*}\right)\\ =\left(\lambda_{0j}^{*}+\xi \lambda_{1j}^{*}\right){\left(\Phi_{0i}^{*}+\xi \Phi_{1i}^{*}\right)}^{T}M\left(\Phi_{0j}^{*}+\xi \Phi_{1j}^{*}\right)-{\left(\Phi_{0i}^{*}+\xi \Phi_{1i}^{*}\right)}^{T}K\left(\Phi_{0j}^{*}+\xi \Phi_{1j}^{*}\right) \end{array}$$

Then Equation (24) and Equation (25) can be rewritten in matrix format, respectively, as

(26) $$\left[\begin{array}{cc} C^{\left(0\right)} & E^{\left(0\right)} \end{array}\right]\gamma^{\left(1\right)}+\left[\begin{array}{cc} C^{\left(1\right)} & E^{\left(1\right)} \end{array}\right]\gamma^{\left(0\right)}=f^{\left(1\right)}$$

(27) $$\left[\begin{array}{cc} C_{†}^{{}^{\left(0\right)}} & E_{†}^{{}^{\left(0\right)}} \end{array}\right]\gamma^{\left(1\right)}+\left[\begin{array}{cc} C_{†}^{{}^{\left(1\right)}} & E_{†}^{{}^{\left(1\right)}} \end{array}\right]\gamma^{\left(0\right)}=f_{†}^{{}^{\left(1\right)}}$$

where $C^{\left(1\right)}={\left(\Phi_{i}\right)}^{T}K_{n}\left(\Phi_{1j}^{*}\right)$, $E^{\left(1\right)}=-\lambda_{0j}^{*}{\left(\Phi_{i}\right)}^{T}M_{n}\left(\Phi_{1j}^{*}\right)$, $f^{\left(1\right)}=\left(\lambda_{0j}^{*}-\lambda_{i}\right){\left(\Phi_{i}\right)}^{T}M\Phi_{1j}^{*}+\lambda_{1j}^{*}{\left(\Phi_{i}\right)}^{T}M\Phi_{0j}^{*}$, $C_{†}^{{}^{\left(1\right)}}={\left(\Phi_{0i}^{*}\right)}^{T}K_{n}\Phi_{1j}^{*}+{\left(\Phi_{1i}^{*}\right)}^{T}K_{n}\Phi_{0j}^{*}$, $E_{†}^{{}^{\left(1\right)}}=-\lambda_{0j}^{*}{\left(\Phi_{0i}^{*}\right)}^{T}M_{n}\Phi_{1j}^{*}-\lambda_{0j}^{*}{\left(\Phi_{1i}^{*}\right)}^{T}M_{n}\Phi_{0j}^{*}-\lambda_{1j}^{*}{\left(\Phi_{0i}^{*}\right)}^{T}M_{n}\Phi_{0j}^{*}$, $f_{†}^{{}^{\left(1\right)}}=\lambda_{1j}^{*}{\left(\Phi_{0i}^{*}\right)}^{T}M\Phi_{0j}^{*}+\lambda_{0j}^{*}{\left(\Phi_{0i}^{*}\right)}^{T}K\Phi_{1j}^{*}+\lambda_{0j}^{*}{\left(\Phi_{1i}^{*}\right)}^{T}M\Phi_{0j}^{*}-{\left(\Phi_{0i}^{*}\right)}^{T}K\Phi_{1j}^{*}-{\left(\Phi_{1i}^{*}\right)}^{T}K\Phi_{0j}^{*}$ and $\gamma^{\left(1\right)}={\left[\begin{array}{cc} \alpha^{\left(1\right)} & \beta^{\left(1\right)} \end{array}\right]}^{T}$.

Combining Equation (26) with (27) together, we have

(28) $$\left[\begin{array}{cc} C_{I}^{{}^{\left(0\right)}} & E_{I}^{{}^{\left(0\right)}} \end{array}\right]\gamma^{\left(1\right)}+\left[\begin{array}{cc} C_{I}^{{}^{\left(1\right)}} & E_{I}^{{}^{\left(1\right)}} \end{array}\right]\gamma^{\left(0\right)}=f_{I}^{{}^{\left(1\right)}}$$

where $C_{I}^{{}^{\left(0\right)}}=\left[\begin{array}{c} C^{\left(0\right)}\\ C_{†}^{{}^{\left(0\right)}} \end{array}\right]$, $E_{I}^{{}^{\left(0\right)}}=\left[\begin{array}{c} E^{\left(0\right)}\\ E_{†}^{{}^{\left(0\right)}} \end{array}\right]$, $C_{I}^{{}^{\left(1\right)}}=\left[\begin{array}{c} C_{}^{{}^{\left(1\right)}}\\ C_{†}^{{}^{\left(1\right)}} \end{array}\right]$, $E_{I}^{{}^{\left(1\right)}}=\left[\begin{array}{c} E_{}^{{}^{\left(1\right)}}\\ E_{†}^{{}^{\left(1\right)}} \end{array}\right]$ and $f_{I}^{{}^{\left(0\right)}}=\left[\begin{array}{c} f_{}^{{}^{\left(0\right)}}\\ f_{†}^{{}^{\left(0\right)}} \end{array}\right]$.

Note that the elements of the coefficient vectors $\alpha^{\left(1\right)}$ and $\beta^{\left(1\right)}$ in $\gamma^{\left(1\right)}$ are $\alpha_{n1}$ and $\beta_{n1}$ (*n* = 1, …, $N_{e}$), respectively. Afterwards, considering the second-order power polynomial base $\xi^{2}$, one can have

(29) $$\left[\begin{array}{cc} C^{\left(0\right)} & E^{\left(0\right)} \end{array}\right]\gamma^{\left(2\right)}+\left[\begin{array}{cc} C^{\left(1\right)} & E^{\left(1\right)} \end{array}\right]\gamma^{\left(1\right)}=f_{}^{{}^{\left(2\right)}}$$

(30) $$\left[\begin{array}{cc} C_{†}^{{}^{\left(0\right)}} & E_{†}^{{}^{\left(0\right)}} \end{array}\right]\gamma^{\left(2\right)}+\left[\begin{array}{cc} C_{†}^{{}^{\left(1\right)}} & E_{†}^{{}^{\left(1\right)}} \end{array}\right]\gamma^{\left(1\right)}+\left[\begin{array}{cc} C_{†}^{{}^{\left(2\right)}} & E_{†}^{{}^{\left(2\right)}} \end{array}\right]\gamma^{\left(0\right)}=f_{†}^{{}^{\left(2\right)}}$$

where $C_{†}^{{}^{\left(2\right)}}={\left(\Phi_{1i}^{*}\right)}^{T}K_{n}\Phi_{1j}^{*}$, $E_{†}^{{}^{\left(2\right)}}=-\lambda_{1j}^{*}{\left(\Phi_{0i}^{*}\right)}^{T}M_{n}\Phi_{1j}^{*}-\lambda_{1j}^{*}{\left(\Phi_{1i}^{*}\right)}^{T}M_{n}\Phi_{0j}^{*}-\lambda_{0j}^{*}{\left(\Phi_{1i}^{*}\right)}^{T}M_{n}\Phi_{1j}^{*}$, $f^{\left(2\right)}=\lambda_{1j}^{*}{\left(\Phi_{i}\right)}^{T}M\Phi_{1j}^{*}$, $f_{†}^{{}^{\left(2\right)}}=\lambda_{1j}^{*}{\left(\Phi_{0i}^{*}\right)}^{T}M\Phi_{1j}^{*}+\lambda_{1j}^{*}{\left(\Phi_{1i}^{*}\right)}^{T}M\Phi_{0j}^{*}+\lambda_{0j}^{*}{\left(\Phi_{1i}^{*}\right)}^{T}M\Phi_{1j}^{*}-{\left(\Phi_{1i}^{*}\right)}^{T}K\Phi_{1j}^{*}$ and $\gamma^{\left(2\right)}={\left[\begin{array}{cc} \alpha^{\left(2\right)} & \beta^{\left(2\right)} \end{array}\right]}^{T}$.

Afterwards, one can rewrite Equations (29) and (30) together as a new algebraic equation, as

(31) $$\left[\begin{array}{cc} C_{I}^{{}^{\left(0\right)}} & E_{I}^{{}^{\left(0\right)}} \end{array}\right]\gamma^{\left(2\right)}+\left[\begin{array}{cc} C_{I}^{{}^{\left(1\right)}} & E_{I}^{{}^{\left(1\right)}} \end{array}\right]\gamma^{\left(1\right)}+\left[\begin{array}{cc} C_{I}^{{}^{\left(2\right)}} & E_{I}^{{}^{\left(2\right)}} \end{array}\right]\gamma^{\left(0\right)}=f_{I}^{{}^{\left(2\right)}}$$

where $C_{I}^{{}^{\left(2\right)}}=\left[\begin{array}{c} O\\ C_{†}^{{}^{\left(2\right)}} \end{array}\right]$, $E_{I}^{{}^{\left(2\right)}}=\left[\begin{array}{c} O\\ E_{†}^{{}^{\left(2\right)}} \end{array}\right]$ and $f_{I}^{{}^{\left(2\right)}}=\left[\begin{array}{c} O\\ f_{†}^{{}^{\left(2\right)}} \end{array}\right]$; additionally, O means a zero matrix or vector.

Following the above steps, the third and fourth order expanding coefficients can be determined by

(32) $$\left[\begin{array}{cc} C_{I}^{{}^{\left(0\right)}} & E_{I}^{{}^{\left(0\right)}} \end{array}\right]\gamma^{\left(3\right)}=f_{I}^{{}^{\left(3\right)}}-\left[\begin{array}{cc} C_{I}^{{}^{\left(1\right)}} & E_{I}^{{}^{\left(1\right)}} \end{array}\right]\gamma^{\left(2\right)}-\left[\begin{array}{cc} C_{I}^{{}^{\left(2\right)}} & E_{I}^{{}^{\left(2\right)}} \end{array}\right]\gamma^{\left(1\right)}-\left[\begin{array}{cc} O & E_{I}^{{}^{\left(3\right)}} \end{array}\right]\gamma^{\left(0\right)}$$

(33) $$\left[\begin{array}{cc} C_{I}^{{}^{\left(0\right)}} & E_{I}^{{}^{\left(0\right)}} \end{array}\right]\gamma^{\left(4\right)}=-\left[\begin{array}{cc} C_{I}^{{}^{\left(1\right)}} & E_{I}^{{}^{\left(1\right)}} \end{array}\right]\gamma^{\left(3\right)}-\left[\begin{array}{cc} C_{I}^{{}^{\left(2\right)}} & E_{I}^{{}^{\left(2\right)}} \end{array}\right]\gamma^{\left(2\right)}-\left[\begin{array}{cc} O & E_{I}^{{}^{\left(3\right)}} \end{array}\right]\gamma^{\left(1\right)}$$

where $E_{†}^{{}^{\left(3\right)}}=\lambda_{1j}^{*}{\left(\Phi_{1i}^{*}\right)}^{T}M_{n}\Phi_{1j}^{*}$,.$f_{†}^{{}^{\left(3\right)}}=\lambda_{1j}^{*}{\left(\Phi_{1i}^{*}\right)}^{T}M\Phi_{1j}^{*}$, $E_{I}^{{}^{\left(3\right)}}=\left[\begin{array}{c} O\\ E_{†}^{{}^{\left(3\right)}} \end{array}\right]$, $f_{I}^{{}^{\left(3\right)}}=\left[\begin{array}{c} O\\ f_{†}^{{}^{\left(3\right)}} \end{array}\right]$, $\gamma^{\left(3\right)}={\left[\begin{array}{cc} \alpha^{\left(3\right)} & \beta^{\left(3\right)} \end{array}\right]}^{T}$ and $\gamma^{\left(4\right)}={\left[\begin{array}{cc} \alpha^{\left(4\right)} & \beta^{\left(4\right)} \end{array}\right]}^{T}$.

By solving Equations (23), (28), and (31)–(33), the vector $\gamma^{\left(0\right)}$–$\gamma^{\left(4\right)}$ can be obtained. Similarly, the higher order of expansion coefficient vectors can be achieved. Then, based on the perturbation solutions of $\alpha_{n1}^{}$ (*i* = 1, 2, …) and $\beta_{n1}^{}$ (*i* = 1, 2, …), a Galerkin projection method is employed to increase the accuracy of solutions of the updated coefficients. The key steps of the projection method are demonstrated in the following.

Using Equations (23) and (31)–(33), Equations (17) and (18) can be rewritten as

(34) $$\begin{array}{l} \left[\begin{array}{cc} C_{I}^{{}^{\left(0\right)}} & E_{I}^{{}^{\left(0\right)}} \end{array}\right](\gamma^{\left(0\right)}+\gamma^{\left(1\right)}\xi +\gamma^{\left(2\right)}\xi^{2}+\gamma^{\left(3\right)}\xi^{3}+\cdots )+\left[\begin{array}{cc} C_{I}^{{}^{\left(1\right)}} & E_{I}^{{}^{\left(1\right)}} \end{array}\right](\gamma^{\left(0\right)}+\gamma^{\left(1\right)}\xi +\gamma^{\left(2\right)}\xi^{2}+\gamma^{\left(3\right)}\xi^{3}+\cdots )\xi \\ +\left[\begin{array}{cc} C_{I}^{{}^{\left(2\right)}} & E_{I}^{{}^{\left(2\right)}} \end{array}\right](\gamma^{\left(0\right)}+\gamma^{\left(1\right)}\xi +\gamma^{\left(2\right)}\xi^{2}+\gamma^{\left(3\right)}\xi^{3}+\cdots )\xi^{2}=f_{I}^{{}^{\left(0\right)}}+f_{I}^{{}^{\left(1\right)}}\xi +f_{I}^{{}^{\left(2\right)}}\xi^{2}+f_{I}^{{}^{\left(3\right)}}\xi^{3}\; \end{array}$$

Reassume the updated coefficient vector γ as

(35) $$\gamma =\sum_{i=0}^{m}\eta_{i}\Gamma_{i}$$

where $\eta_{i}$ (*i* = 0, 1, …, *m*) mean the coefficients of the new expansion bases $\Gamma_{i}$, which denote the vector bases $\gamma^{\left(0\right)}$, $\gamma^{\left(1\right)}\xi$, $\gamma^{\left(2\right)}\xi^{2}$, $\gamma^{\left(3\right)}\xi^{3}$ and so on for the Galerkin projection [30]. *m* is the number of the expansion order.

To determine the coefficients $\eta_{i}$ of the updated coefficient vector, substituting Equation (35) into Equation (34) and premultiplying both sides of Equation (34) by ${\left(\left[\begin{array}{cc} C_{I}^{{}^{\left(0\right)}} & E_{I}^{{}^{\left(0\right)}} \end{array}\right]\Gamma_{k}\right)}^{T}$ (*k* = 0, 1, …, *m*), the deterministic algebraic equations are obtained as

(36) $$\sum_{i=0}^{m}{\bar{CE}}_{ki}^{\left(0\right)}\eta_{i}+\sum_{i=0}^{m-1}{\bar{CE}}_{ki}^{\left(1\right)}\eta_{i}+\sum_{i=0}^{m-2}{\bar{CE}}_{ki}^{\left(2\right)}\eta_{i}={\bar{f}}_{}^{{}_{k}}\;(k=0,1\cdots m)$$

where ${\bar{CE}}_{ki}^{\left(0\right)}=<{\left(\left[\begin{array}{cc} C_{I}^{{}^{\left(0\right)}} & E_{I}^{{}^{\left(0\right)}} \end{array}\right]\Gamma_{k}\right)}^{T}\left[\begin{array}{cc} C_{I}^{{}^{\left(0\right)}} & E_{I}^{{}^{\left(0\right)}} \end{array}\right]\Gamma_{i}>$, ${\bar{CE}}_{ki}^{\left(1\right)}=<{\left(\left[\begin{array}{cc} C_{I}^{{}^{\left(0\right)}} & E_{I}^{{}^{\left(0\right)}} \end{array}\right]\Gamma_{k}\right)}^{T}\left[\begin{array}{cc} C_{I}^{{}^{\left(1\right)}} & E_{I}^{{}^{\left(1\right)}} \end{array}\right]\Gamma_{i}\xi >$, ${\bar{CE}}_{ki}^{\left(2\right)}=<{\left(\left[\begin{array}{cc} C_{I}^{{}^{\left(0\right)}} & E_{I}^{{}^{\left(0\right)}} \end{array}\right]\Gamma_{k}\right)}^{T}\left[\begin{array}{cc} C_{I}^{{}^{\left(2\right)}} & E_{I}^{{}^{\left(2\right)}} \end{array}\right]\Gamma_{i}\xi^{2}>$, ${\bar{f}}_{}^{{}_{k}}=\sum_{i=0}^{2}<{\left(\left[\begin{array}{cc} C_{I}^{{}^{\left(0\right)}} & E_{I}^{{}^{\left(0\right)}} \end{array}\right]\Gamma_{k}\right)}^{T}f^{\left(i\right)}\xi^{i}>$, and <⋅> is a mathematical expectation operator. It is clear that ${\bar{CE}}_{ki}^{\left(0\right)}$, ${\bar{CE}}_{ki}^{\left(1\right)}$, ${\bar{CE}}_{ki}^{\left(2\right)}$ and ${\bar{f}}_{}^{{}_{k}}$ are scalars. Equations (36) involve *m* + 1 undetermined coefficients $\eta_{i}$. Then, by solving the (m+1)×(m+1) linear algebraic equations, the unknown coefficients $\eta_{0}$, $\eta_{1}$ … $\eta_{m}$ can be determined.

From the above process, it can be observed that the proposed updating method effectively combines the hybrid perturbation method with the ICMCM method together, which is named as HPG-ICMCM in this paper. Given that $\left[\begin{array}{cc} C_{I}^{{}^{\left(0\right)}} & E_{I}^{{}^{\left(0\right)}} \end{array}\right]$ and $\left[\begin{array}{cc} C_{I}^{{}^{\left(1\right)}} & E_{I}^{{}^{\left(1\right)}} \end{array}\right]$ in Equation (32), Equation (33) and Equation (36) are replaced by $\left[\begin{array}{cc} C^{\left(0\right)} & E^{\left(0\right)} \end{array}\right]$ and $\left[\begin{array}{cc} C^{\left(1\right)} & E^{\left(1\right)} \end{array}\right]$, respectively, the vectors $\gamma^{\left(0\right)}$–$\gamma^{\left(4\right)}$ and γ may be recursively obtained by solving Equations (21), (26), (29), (32), (33) and (36). In this way, the proposed method will degenerate into the HPG-CMCM method.

**Figure 1:**
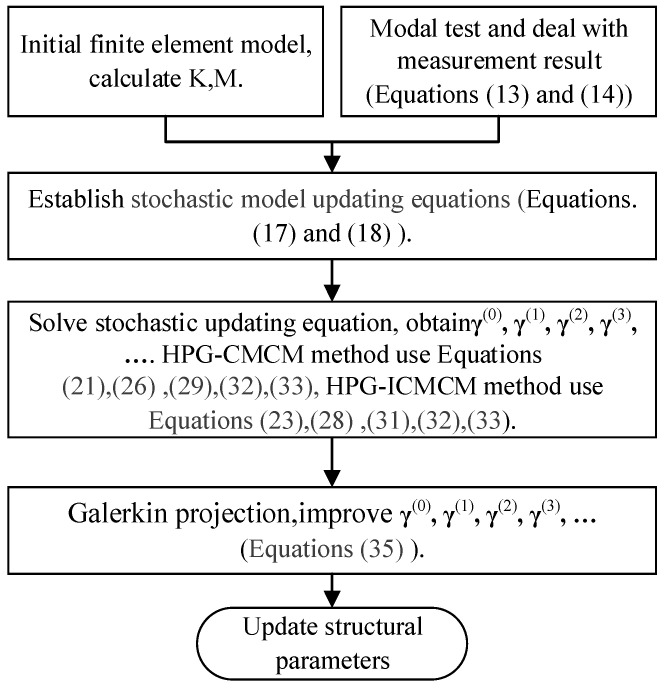
Flowchart of stochastic model updating by means of the HPG-ICMCM method.

It is worth mentioning that for actual structures, the rotational modes are often difficult to measure. Additionally, due to the limitation of measurement conditions, the complete displacement modes are not always achievable. Therefore, in this paper, the modal expansion method in [12] is used to get the mean $\Phi_{0j}^{*}$ and the standard deviation $\Phi_{1j}^{*}$ of the complete modal shape of the *j*th measured mode. At the same time, in solving Equation (23), the singular value truncation regularization technique is taken to avoid the ill-conditioned equations. The flow chart of the proposed method in this paper is shown in Figure 1.

## 3. Numerical Study: A Two-Span Continuous Beam

**Figure 2:**
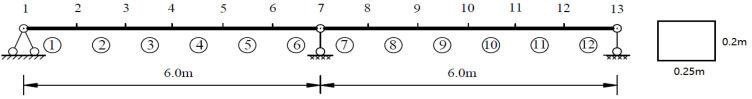
A two-span continuous beam.

This section studies the model updating of a two-span beam. The length of span and the section of the beam are drawn in Figure 2. The FE model of the simulated continuous beam is composed of 12 identical Euler–Bernoulli beam elements. Every node in element contains two degrees of freedom, a vertical displacement and a rotation angle. For the initial beam model, it is assumed that the elastic modulus and density are 2.8 × l0^10^ Pa and 2.5 × l0^3^ kg/m^3^, respectively.

It is also supposed that the actual mass values of 2nd, 5th and 10th elements are reduced by 40%, 30% and 20%, At the same time, the elastic moduli of the 3rd, 5th, 9th, 10th and 11th elements decrease by 30%, 40%, 35%, 30% and 20%, respectively, and the mass and elastic modulus of other elements are the same as the initial values. The elastic modulus and mass of the 12 elements are selected as the updated parameters. The updated parameters involving the elastic modulus are numbered from 1 to 12 from left to right, and accordingly, the mass number is from 13 to 24. In other words, the total number of the updated parameters is 24. The simulated modal data of the beam with deterministic reduced parameters are regarded as the results of the true model. It is assumed that the measured modal data are random, and the coefficients of variation (COV) of the measured modal data is 0.02. The modal data are of Beta distributions. The three methods, including the proposed HPG-ICMCM method, the HPG-CMCM method and the corresponding Monte Carlo simulation methods (MCS-CMCM and MCS-ICMCM), are used to update this continuous beam. For the MCS-CMCM and MCS-ICMCM methods, 1 × 10^6^ samples are produced by the assumption of stochastic measurement data, and the simulation results are utilized to verify the updating effectiveness of the HPG-ICMCM method as a benchmark.

First of all, assume that the first six measured frequencies and displacement modal shapes are measured. The complete forms of measured modal shapes are obtained by modal expansion. In regard to the initial simulation model, the first six order modal data are calculated. Therefore, for the HPG-ICMCM method, the dimension of updating equations reaches 54. When the regularization technique, which is demonstrated in [12], is applied to Equation (23), the first 22 larger singular values are left, and the two minimum ones are removed. The same technique is also used in the other three methods.

**Figure 3:**
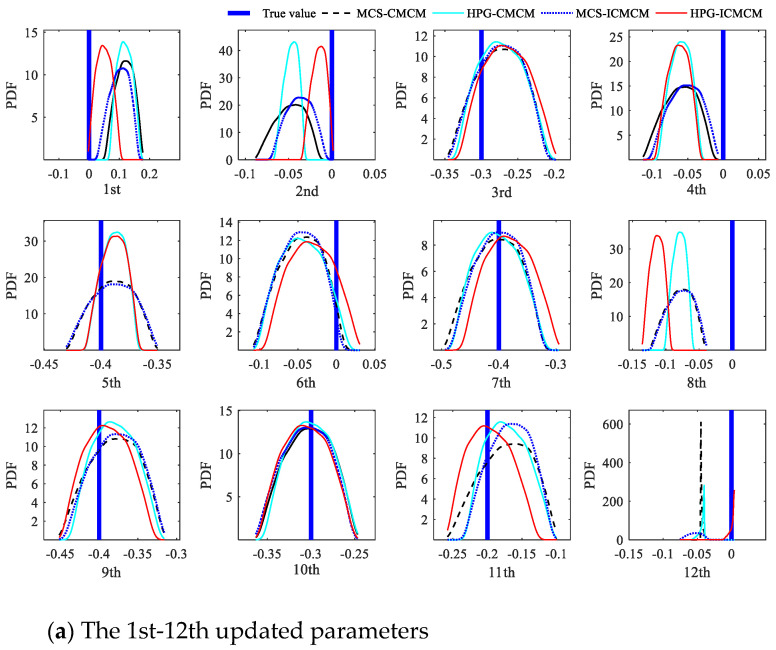

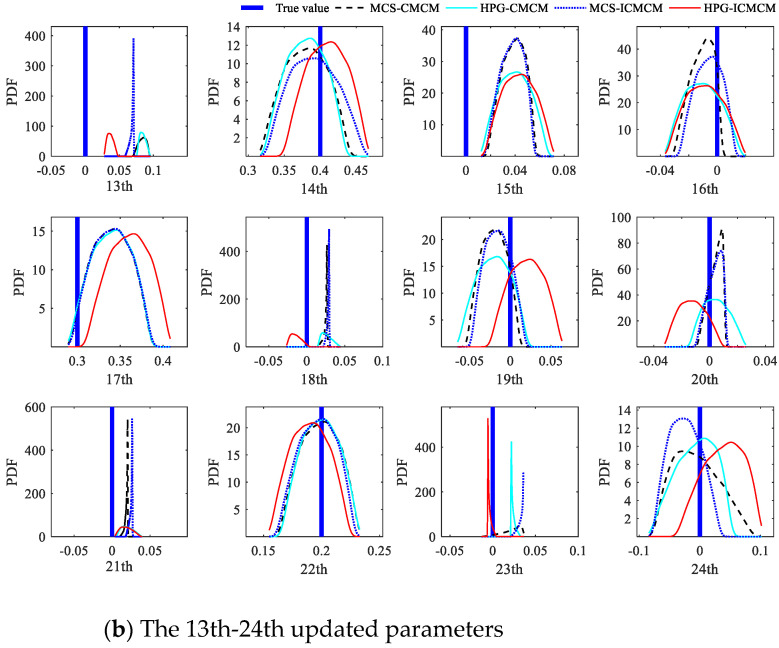
The probability density functions of the 24 updated parameters.

The probability density functions (PDF) of the updated parameters of all elements in the beam are shown in Figure 3. From Figure 3, it is found that the PDF curves of the updated parameters determined using the HPG-ICMCM method closely approach the PDF curves by the MCS-ICMCM method, and the results from the HPG-CMCM method also are in a good agreement with those by the MCS-CMCM method. This phenomenon demonstrates that the updating effectiveness of the proposed HPG-ICMCM method and the HPG-CMCM method is satisfied. Meanwhile it is observed from Figure 3 that generally the PDFs of the updated parameters using the HPG-ICMCM method are closer to the true values of the updated parameters than those by the HPG-CMCM method, which indicates the updating of the proposed HPG-ICMCM method is more accurate than that of the HPG-CMCM method.

**Figure 4:**
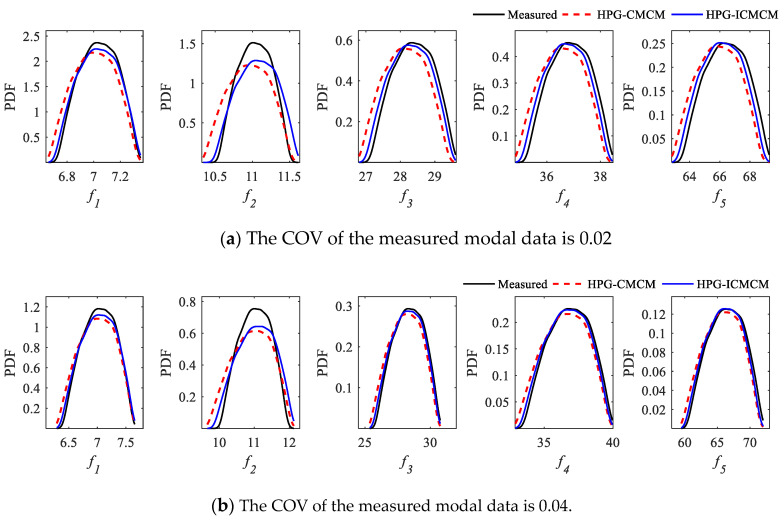
PDFs of the first five updated frequencies.

Furthermore, using Equation (1) and replacing the original initial parameters by the updated parameters, the PDFs of updated frequencies of the continuous beam can be calculated by the Monte Carlo simulation. The PDF curves of the first five updated frequencies of the beam are shown in Figure 4a. It can be seen from Figure 4a that the PDF curves of the first five updated frequencies calculated by the suggested method and the results by the HPG-CMCM method coincide with each other, and the PDF curves of both methods are very consistent with those of the measurement frequencies. This finding further confirms that the updating effect of the proposed HPG-ICMCM method is very good.

If one increases the COV of the measured modal data from 0.02 to 0.04, the PDF curves of the first five updated frequencies can also be obtained by the HPG-ICMCM method and the HPG-CMCM method, which are plotted in Figure 4b. From Figure 4b, it can be observed that even if the COV of the measured modal data increases to 0.04, and the measurement data have a relatively large fluctuation, the PDF curves of the updated frequencies by the two methods are still close to those measured frequencies. This finding shows that the proposed updating method can effectively deal with large uncertainty in measured data. On the other hand, the computational time of the proposed method is 12 s on a personal computer of Dell Inspiron 7391 with an Intel core I7-10510U CPU and a 8 GB memory, whereas the computational time of the MCS-ICMCM method and the MCS-CMCM method based on 1 × 10^6^ samples is more than 1800 s, which shows that the proposed method is of high efficiency.

Now assume that for the continuous beam, only few modes can be measured. It is pointed out in [22] that when the number of measurement mode is limited, the deterministic CMCM method may result in the instability of solutions of updating equations. Next, the impact of the limited measured modes on the updating results will be studied using the proposed updating method. In the following, it is assumed that only the first four, three and two modes are measured, respectively, and correspondingly, the first six, three and four modes of the initial model are used, respectively, so that the three cases involving the combination of measurement and calculation modes are designed as shown in Table 1. Table 1 also lists the number of updating equations in the three cases. Further it is assumed that compared with the initial values of elastic modulus, the true elastic moduli of the 3rd, 5th, 9th, 10th and 11th elements decrease by 30%, 40%, 35%, 30% and 20%, respectively. The mass of the beam elements remains unchanged.

**Figure 5:**
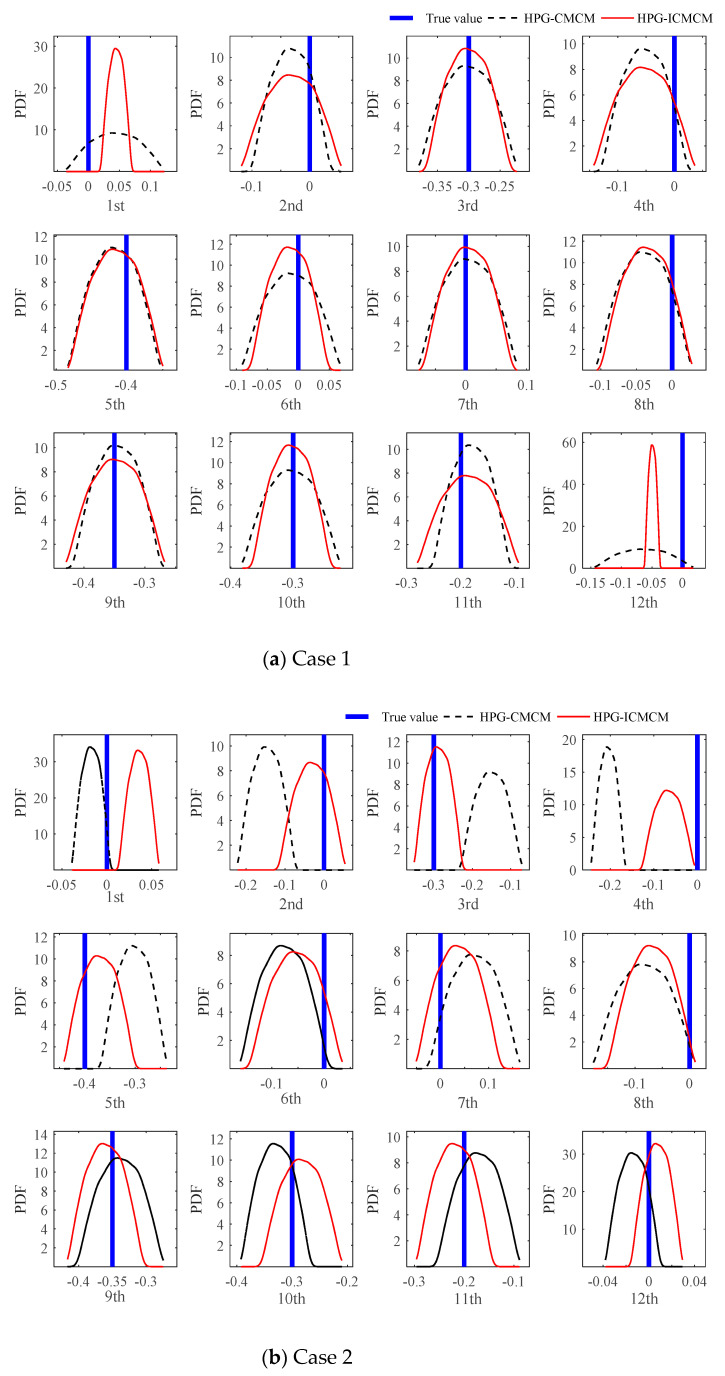

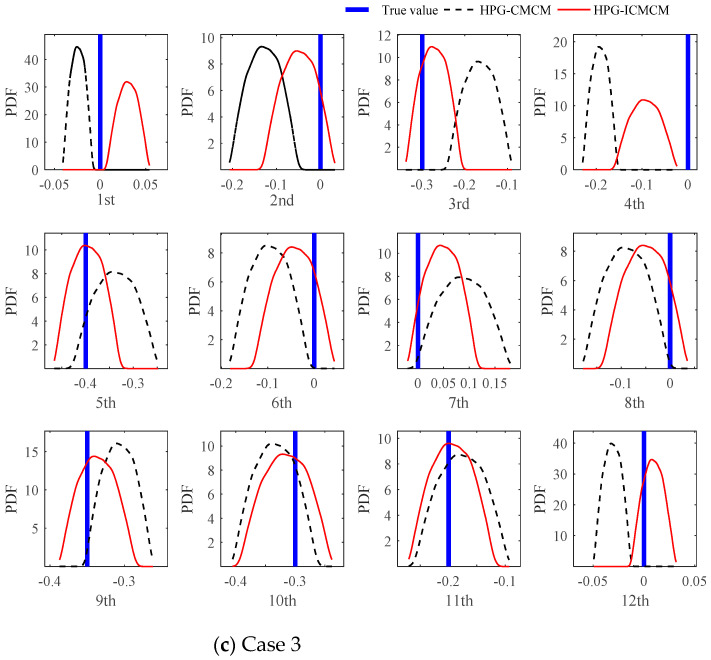
PDFs of the updated parameters of all elements in the three cases.

The proposed HPG-ICMCM method and the HPG-CMCM method are performed to update the continuous beam in the three cases and obtain the PDFs of the updated parameters, which are the elastic moduli of the 12 elements, as shown in Figure 5. It is found from Figure 5a that when the ranks of the submatrix $C_{}^{{}^{\left(0\right)}}$ and that of $C_{I}^{{}^{\left(0\right)}}$ are full and identical, the PDFs of the updated parameters by the two methods agree with each other very well, and they are very close to the true values of the updated parameters. Furthermore, it can be observed from Figure 5b,c that as the number of measurement modes decreases, the results from the HPG-CMCM method become worse. However, the proposed HPG-ICMCM method still has very good updating effectiveness. The reason why this phenomenon occurs comes from the increment of the dimension of updating equations, which leads to the full rank of the submatrix $C_{I}^{{}^{\left(0\right)}}$.

## 4. Experimental Verification

**Figure 6:**
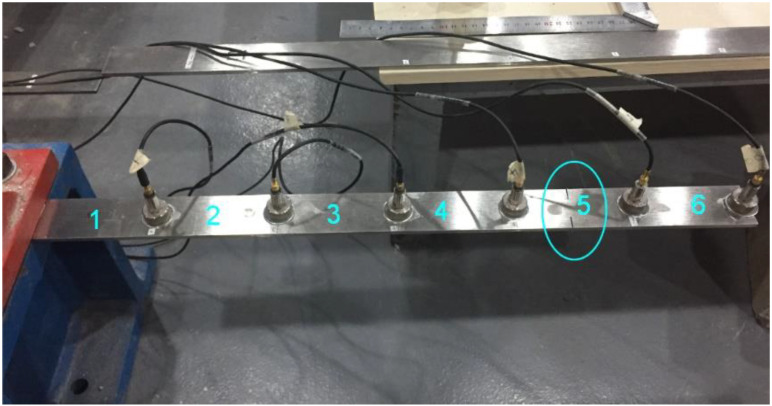
A steel cantilever beam.

A steel cantilever beam, which is shown in Figure 6, was built in a laboratory in Wuhan University of Technology. The length and section size of the beam were 570 mm and 40 × 4 mm^2^, respectively. The parameters of material properties, density, elastic modulus and Poisson’s ratio, were 7800 kg/m^3^, 210 GPa and 0.33, respectively. One end of the beam was fixed. In dynamic tests, the beam was divided into six elements and six accelerometers were uniformly distributed on the six elements of the beam, as shown in Figure 6. The mass of both each accelerometer and its auxiliary magnet was 25.1 g, accounting for 18% of the mass of each element of the beam. The above assigned structural parameters were used as the calculation parameters of the initial model. In the experiment, the saw kerf was made on Element 5 and the depth of the kerf accounts for 50% of the width of cross section of the beam. The loss of mass caused by the kerf was ignored.

**Figure 7:**
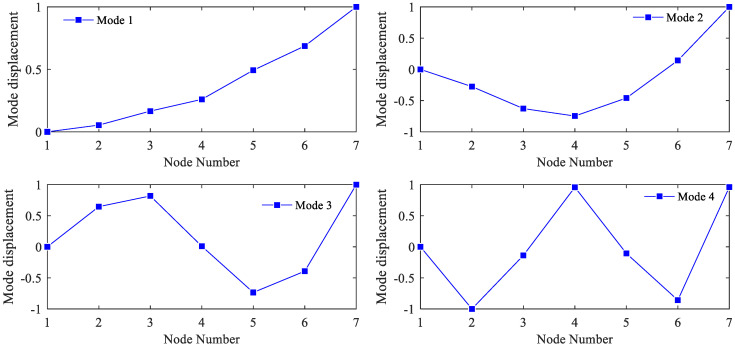
Means of the first four order measured modal shapes.

After the acceleration histories at the six measurement points were recorded, the Enhanced Frequency Domain Decomposition modal identification technique [31] was used to identify the modal data of the experimental beam. The mean values of the first four measured frequencies were 8.25, 52.56, 141.90 and 279.49 Hz, respectively. The means of the first four modal shapes are shown in Figure 7. After being expanded, the first three measured displacement modal shapes and their corresponding measurement frequencies were used for updating. Meanwhile, the first three simulated modal shapes and frequencies of the initial model were also selected. In the process of updating the model, the elastic moduli and mass values of the 6 elements were chosen as updated parameters.

**Figure 8:**
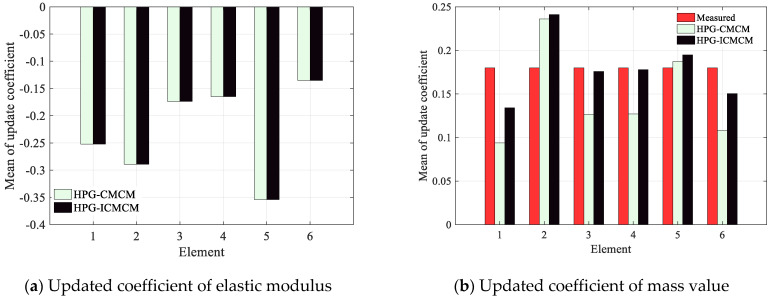
The means of the updated coefficients of the 12 updated parameters including the six elastic moduli and the six mass values.

The proposed HPG-ICMCM method and the HPG-CMCM method were used for the model updating of the beam. The means of updated coefficients of the 12 updated parameters, including 6 elastic moduli and 6 mass values, are plotted in Figure 8. It can be seen from Figure 8a that the means of updated coefficients of 6 elastic moduli by the proposed HPG-ICMCM method are in a very good agreement with those of the HPG-CMCM method. The means of updated coefficients of the 6 elastic moduli are negative, which means that the elastic moduli of the experimental beam are less than those of the initial model. It is also found that the mean of updated coefficient at the cut kerf reaches −0.35, and it is obviously less than the values at other positions of the beam, which indicates that the degradation of stiffness of the 5th element occurs due to the cut kerf. In addition, from Figure 8b, it is seen that for the mean values of mass of the six elements, the results at 1st, 3rd, 4th and 6th element from the proposed method are better than that by the HPG-CMCM method compared with the measured mass values.

**Figure 9:**
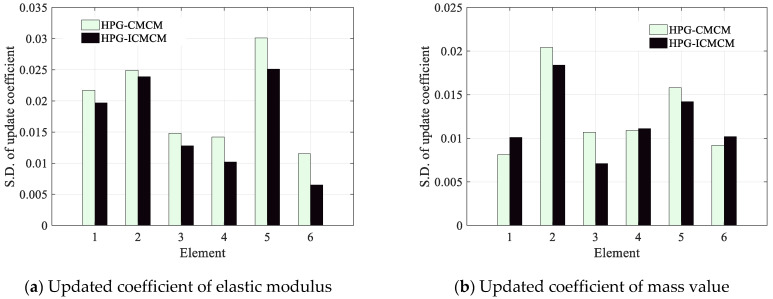
The standard deviations of the updated coefficients of the 12 updated parameters including the six elastic moduli and the six mass values.

The standard deviations of updated coefficients of the 12 updated parameters, including 6 elastic moduli and 6 mass values, are plotted in Figure 9. It can be seen from Figure 9 that as a whole, the standard deviations of updated coefficients of the 12 updated parameters by the proposed method are less that those by the HPG-CMCM method. It can be derived that the COVs of updated coefficients of the 12 updated parameters are about 0.1, and actually the COVs of the measured frequencies are only 0.01 or so, which are obviously less than that of the updated parameters. This finding indicates that the small uncertainty of measured frequencies will cause a large fluctuation of the updated coefficients and the fluctuation of updated coefficients caused by the HPG-ICMCM method is less than that by the HPG-CMCM method.

**Figure 10:**
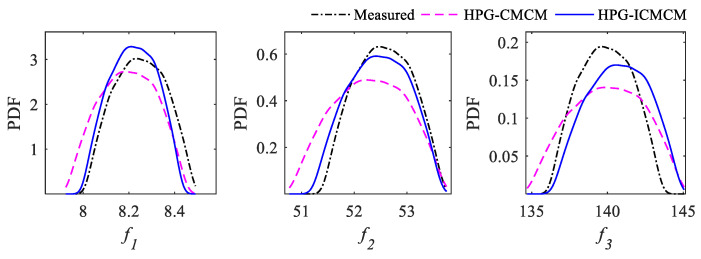
PDFs of the first three updated frequencies.

To further testify the updating effectiveness of the proposed method, the PDFs of updated frequencies of the experimental beam were simulated by using the 12 updated parameters, and the results are drawn in Figure 10. It can be found that the PDF curves of the HPG-ICMCM method are in a very good agreement with those of the measured frequencies, the PDF curves of the updated frequencies calculated by the proposed HPG-ICMCM method are closer to the PDF curves of the measured frequencies than those by the HPG-CMCM method.

## 5. Conclusions

A new stochastic FE model updating method, the HPG-ICMCM method, was proposed in this paper. Through combining with the hybrid perturbation-Galerkin method, this proposed method extends the deterministic ICMCM method to the stochastic domain, mainly including the establishment and solution of the stochastic model updating equations. This new method sufficiently uses the advantage of the ICMCM method, which can deal with the limited measurement data problem, and that of which the hybrid perturbation Galerkin method can accurately and efficiently solve stochastic updating equations.

The numerical example of a continuous beam shows that the HPG-ICMCM method can effectively cope with relatively large uncertainty in measured data, and the computational efficiency of this new method is several orders of magnitude higher than that of the Monte Carlo simulation method. When the measurement data are limited such as only first few modal data or in the case of rank deficiency, the HPG-ICMCM method has a better updating effectiveness than the HPG-CMCM method compared with the assumed real values of updated parameters. In the experimental study of a cantilever steel beam, the proposed HPG-ICMCM method can still match with the measurement results very well and the fluctuation of random updated parameters by the HPG-ICMCM method is less than that by the HPG-CMCM method, which illustrates that the updating effectiveness of the proposed method is satisfied. The deterministic ICMCM model updating method needs to use large samples for Monte Carlo simulation in solving random problems, which takes a long time. In contrast, the method proposed in this paper can deal with the problem of stochastic model updating well, especially when the measurement variability is large the method proposed in this paper can get accurate stochastic update parameters efficiently. In the follow-up research, the application of the proposed method on actual engineering structures will be discussed since the proposed new method does not limit the scale of FE models updated.

## Figures and Tables

**Table 1:** The number of measured modes and updating equations under the six cases.

Case No.	Number of Modes	Method	Rank	Number of Updating Equations
Case 1	*N_c_* = 6, *N_m_* = 4	CMCM	$\mathrm{Rank}\;(C_{}^{{}^{\left(0\right)}})$ = 12	24
		ICMCM	$\mathrm{Rank}\;(C_{I}^{{}^{\left(0\right)}})$ = 12	40
Case 2	*N_c_* = 3, *N_m_* = 3	CMCM	$\mathrm{Rank}\;(C_{}^{{}^{\left(0\right)}})$ = 9	9
		ICMCM	$\mathrm{Rank}\;(C_{I}^{{}^{\left(0\right)}})$ = 12	18
Case 3	*N_c_* = 4, *N_m_* = 2	CMCM	$\mathrm{Rank}\;(C_{}^{{}^{\left(0\right)}})$ = 8	8
		ICMCM	$\mathrm{Rank}\;(C_{I}^{{}^{\left(0\right)}})$ = 12	12

Note: Rank $\left(C_{}^{{}^{\left(0\right)}}\right)$ and Rank $\left(C_{I}^{{}^{\left(0\right)}}\right)$ represent the ranks of the submatrices $C_{}^{{}^{\left(0\right)}}$ in Equation (21) and $C_{I}^{{}^{\left(0\right)}}$ in Equation (23), respectively.

## Data Availability

The data used to support the findings of this study are available from the corresponding author upon request.
